# Immunomodulatory Effects of Zinc and Selenium‐Enriched *Lentinula edodes* Extract: Biochemical Characterization and Bioactivity Relevant to Tuberculosis

**DOI:** 10.1002/fsn3.71736

**Published:** 2026-04-08

**Authors:** Hanif Mughal, Tabussam Tufail, Huma Bader ul Ain, Amara Rasheed, Esther Ugo Alum

**Affiliations:** ^1^ University Institute of Diet and Nutritional Sciences The University of Lahore Lahore Pakistan; ^2^ Department of Food Sciences Government College University Faisalabad Faisalabad Pakistan; ^3^ Department of Research Publications and Extension Kampala International University Kampala Uganda

**Keywords:** fortified, immunomodulatory, mushrooms, Myrin fort, nutrition, tuberculosis

## Abstract

The primary hypothesis of the study was that zinc and selenium supplementation through fortified cookies would enhance the immune response and improve clinical outcomes in tuberculosis patients. For this purpose, a single‐blind randomized control trial was carried out to determine the effect of zinc and selenium on the immunomodulatory parameters of tuberculosis, approved by the Ethics Committee of the University of Lahore, Pakistan. In the first phase, the proximate and minerals profile of shiitake mushroom was analyzed, and functional cookies were developed fortified with zinc and selenium. In the second phase, 120 tuberculosis patients were divided into four groups (T0, T1, T2, and T3) to assess the impact of supplementation on anthropometric, microbiological, and hematological parameters. Meanwhile, results were statistically examined through one‐way ANOVA followed by Duncan's Multiple Range Test and showed significant differences (*p* ≤ 0.05) among treatment groups. In comparison to controls, patients who received Zn and Se‐enriched cookies (T2 and T3) reported significant increases in BMI, hemoglobin, and decreases in CRP and ESR. Additionally, Principal component analysis (PCA) and heat map visualization were generated using R Studio (Version 4.2.2) to identify patterns and correlations among treatments. During tuberculosis infection, those improvements were associated with the antioxidant and immunomodulatory functions of zinc in macrophage activation and selenium‐dependent glutathione peroxidase activity, which are involved in the regulation of oxidative stress. Thus, the results showed that the therapeutic effect of dosage on anthropometric, microbiological, and hematological measurements showed a significant result, indicating that zinc and selenium may help to modulate the immunological response, and potentially improve the body's ability to resist tuberculosis.

**Trial Registration:** ClinicalTrials.gov (Identifier: NCT07180329)

## Graphical abstract

Graphical representation of overall study.

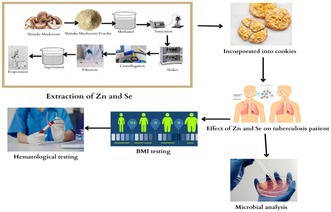

## 1 Introduction

Tuberculosis (TB) remains a leading global cause of death, and its connection with malnutrition forms a complex relationship (Ockenga et al. 2023). TB infection can lead to malnutrition due to increased metabolic demands and reduced nutrient intake, while nutritional deficiencies can exacerbate the disease by impeding crucial immune functions (Yang et al. 2021). Essential micronutrients, particularly trace elements like cobalt (Co), selenium (Se), zinc (Zn), and copper (Cu), play diverse roles, acting as structural components of vitamins, co‐factors in enzymes, catalytic components in various reactions, and essential parts of proteins crucial for immune responses. Given their immunomodulatory functions, trace elements are thought to influence the body's susceptibility to infections, impacting the progression and outcomes of diseases (Rizwan et al. 2025). Meanwhile, plant extracts have shown potential in mitigating liver toxicity by providing antioxidant and anti‐inflammatory effects, protecting liver cells from damage. Studies on both animals and humans suggest improved liver function and reduced toxicity using various medicinal plants (Agarwal et al. 2025).

Mushroom is a macrofungus, noticeable to the naked eye, belonging to the Agaricaceae family with diverse species, of which at least 2000 are considered edible. Mushrooms grow on food sources like decaying wood or soil and have significant nutritional content, comprising 85%–95% moisture, carbohydrates, protein, fat, vitamins, and minerals. Moreover, phenols, flavonoids, polysaccharides, vitamins, carotenoids, ergothioneine, and other mycochemicals with antioxidant action are found in both edible and medicinal mushrooms (Hait et al. 2025). Apart from their culinary uses, mushrooms are an invaluable source of physiologically active substances that have nutraceutical effects. *Lentinula edodes*, commonly known as shiitake, is a widely consumed edible mushroom with recognized nutritional and medicinal properties (Baral 2025). Shiitake is rich in proteins, fats, carbohydrates, vitamins, and minerals, historically recommended for promoting longevity and good health in Chinese medicine. Known for their anticarcinogenic, anti‐inflammatory, antioxidant, antifungal, antibacterial, antiviral, and antithrombotic properties in cardiovascular disorders, shiitake also exhibit prebiotic properties and high nutritive value (Singh et al. 2025). However, before incorporating dried shiitake into the diet, especially as a mineral source, it is crucial to determine their mineral content and their ability to bind micro‐elements (Zakaria et al. 2022).

The balance of trace minerals is vital for various biological functions, impacting the absorption and utilization of nutrients, and is influenced by factors such as dietary intake and digestive system processes (Mehri 2020). Zn, a crucial trace element, is essential for human growth, impacting neurologic, reproductive, and immune systems. By modifying signaling pathways in innate immune cells like neutrophils and macrophages, which are in charge of early pathogen recognition and elimination, zinc plays a critical role in immune regulation (Kim and Lee 2021). Additionally, it controls intracellular signaling pathways (e.g., MAPK and NF‐κB), which in turn control inflammatory reactions, cytokine production, and the activation of immune defense mechanisms against infections. Moreover, functioning in various metalloenzymes and gene expression, Zn deficiency affects innate and acquired immune systems, influencing cells like neutrophils, macrophages, and T cells. It acts as an antioxidant and anti‐inflammatory agent, playing a central role in immune defense and contributing to bactericidal effects on various bacteria, including MTB (Haase and Rink 2014). While necessary for MTB, high Zn concentrations can be toxic. Zn supplementation, especially with vitamin A, has shown improved anti‐TB treatment outcomes and sputum smear conversion. Lower Zn levels are associated with higher C‐reactive protein in TB patients. While Zn's role alongside TB therapy is unclear, evidence suggests it increases ethambutol cytotoxicity and can be used to evaluate treatment outcomes in TB patients (Jones et al. 2024).

Se is essential for the maintenance and development of musculoskeletal, cardiovascular, hormonal, and reproductive systems. Selenoproteins like Se‐dependent glutathione peroxidase (GPx) regulate oxidative stress and inflammation responses, contributing to immune system maintenance and clearance of MTB (Qian et al. 2019). Se's protective role extends to various infections, including viral, bacterial, and fungal, and it is crucial for T lymphocyte proliferation and differentiation, decreasing the risk of TB infection. Se concentration is lower in the serum of TB infected patients, with low levels associated with a higher risk of pulmonary infection (Ciftci et al. 2003). Zinc and selenium, which are essential trace minerals, were identified as critical cofactors in a variety of enzymatic reactions and played a significant role in the regulation of immune function, antioxidant defense, and overall metabolic health (Yatoo et al. 2013). However, the functional contribution of its mineral content to biological activity had been relatively underexplored, despite the fact that previous research had primarily concentrated on the bioactive compounds of Shiitake mushroom, such as phenolics, flavonoids, and polysaccharides (Zhou et al. 2024). The intricate interplay between these minerals and other bioactive metabolites is fundamental to elucidating the mechanisms underlying the health‐promoting properties of *L. edodes* (shiitake). These interactions likely influence the bioavailability and therapeutic efficacy of these components, contributing to the mushroom's demonstrated antioxidant, immunomodulatory, and anti‐inflammatory activities (Xiaoying et al. 2025). Previous studies have demonstrated that in patients with tuberculosis, supplementation with micronutrients, such as vitamin A and selenium, in addition to standard therapy, enhances antioxidant status and reduces oxidative stress. Given the widespread consumption and high consumer acceptability of bakery products, particularly cookies, this study was designed to develop functional cookies fortified with zinc (Zn) and selenium (Se) derived from shiitake mushrooms (*L. edodes*) as a practical, cost‐effective dietary vehicle. The investigation aimed to evaluate the nutritional composition and mineral profile of shiitake mushroom‐fortified formulations, develop Zn‐ and Se‐enriched functional cookies, and assess the impact of this dietary intervention on anthropometric, microbiological, and hematological parameters in tuberculosis (TB) patients undergoing treatment. This study seeks to support a cost‐effective nutritional strategy to complement conventional anti‐TB therapy.

## 2 Materials and Methods

### 2.1 Procurement of Raw Materials

Initially, 1 kg of fresh shiitake was procured from the departmental store of Lahore and placed in a sealed bag (Zip bag). All the research work was performed at the University of Lahore Institute of Food Science and Technology. Shiitake was cut into pieces and shade dried at 28°C–30°C (Relative humidity 55%–65%) for 24 h. (Drying was performed under natural ventilation without forced airflow, allowing free air circulation under shaded conditions). After shade drying for 24 h, approximately 120 g of dried mushroom powder was obtained. The dried mushroom powder was pulverized with a blender (Blantyre (1500 W) Lahore, Pakistan) for 30 s and screened through a 35‐mesh sieve and stored at −20°C until further used. The powdered mushroom sample was extracted using absolute ethanol for 72 h in dark conditions and at a temperature of 30°C. The extract was lyophilized and the crude content was freeze‐dried for future analysis.

### 2.2 Proximate Composition of Shiitake Powder

#### 2.2.1 Moisture Analysis

For the determination of moisture content of sample by using a hot air oven (Memmert Germany), method number 44–15.02 given in AACC (2000). A dried and clean China dish was taken, and 10 g of each raw material was weighed using a digital weighing balance and placed into it. Both samples were dried at 100°C ± 5°C for 24 h in a hot air oven. After the samples were completely dried, they were removed from the oven and cooled in a desiccator to avoid reabsorption of moisture from the environment. Then the sample was again weighed, and the values were noted. The moisture was calculated by the following Equation (1).

(1) $$\text{Moisture}\;\left(\%\right)=\frac{\text{weight of sample before drying}-\text{weight of sample after drying}\;}{\text{weight of sample before drying}}\times 100.$$

#### 2.2.2 Ash Content

Ash content was determined by following the standard procedure given in AACC (2000) and by following the formula given in Equation (2). Thoroughly combined raw materials were gathered in the crucibles, which had been weighed, and charring was carried out on the burner. Then, the samples were transferred to an apparatus muffle furnace (DAIHAN), and the temperature range of 550°C–650°C was used until the samples turned to grayish white color residues. After the cooling process (in desiccators), the crucibles were weighed to avoid the absorption of moisture from the surroundings.

(2) $$\mathrm{Ash}\left(\%\right)=\left(\mathrm{Ash}\;\text{weight}/\text{Sample weight}\right)\times 100$$

#### 2.2.3 Crude Protein Content

Protein content was estimated or analyzed by following the standard AACC (2000) procedure, as given in 46–11 A. In the digestion tube, the sample was digested for three to four hours by using the concentrated H_2_SO_4_ (30 mL) in the digestion mixture of five grams (consisting of CuSO._4_, FeSO._4_, K_2_SO._4_ in the 9:1:90 ratio) until the required color was obtained, which was transparent or the lighter green. The material being digested was then poured into the volumetric flask of 250 mL and the volume was brought to the mark with the help of distilled water. The diluted sample, which was 10 mL, was then distilled with 40% NaOH solution of 10 mL with the help of the distillation apparatus. The gas (ammonia) produced as a result was then collected in the (10 mL of 4%) boric acid, containing the indicator which was methyl red (1–2 drops). Later, the solution was titrated against the 0.1 N H_2_SO_4_ and in the same way, the blank sample was also run. The following equation determined the percentage of nitrogen; no. 3 and crude protein percentage was determined by multiplying % N_2_ with a constant factor of 6.25 as given in Equation (4):

(3) $$\text{Nitrogen}\left(\%\right)=\left(\text{Amount of}\;0.1\;N\;H_{2}{\mathrm{SO}}_{4}\text{used}\times 0.0014\times 250/\mathrm{Wt}.\text{of sample}\right)\times 100$$

(4) $$\text{Crude Protein}\;\left(\%\right)=\text{Nitrogen}\%\times 5.7$$

#### 2.2.4 Crude Fat Content

The crude fat content of raw material was estimated by adopting the procedure as mentioned in AACC (2000) by using the Soxhlet apparatus. Three gram of the dried sample was taken after removal of moisture by using a hot air oven for 24 h. Convert the dried sample into powder and put it into the filter paper by closing the filter paper through the steeper pins. N‐hexane or di‐ethyl ether was used as an organic solvent. The completed 6 to 8 siphon cycles of the Soxhlet apparatus to remove soluble fat from the sample and noted the value by using the formula in terms of percentage as shown in Equation (5).

(5) $$\mathrm{Fat}\;\left(\%\right)=\frac{\text{sample weight before extraction of}\;\mathrm{fat}-\text{sample weight after extraction of}\;\mathrm{fat}\;}{\;\text{Weight of sample before extraction of}\;\mathrm{fat}}\times 100$$

#### 2.2.5 Crude Fiber Content

A sample for crude fiber analysis was collected using the techniques outlined in AACC (2000) Method No. 32–10. The crude fiber content was estimated to be a sample of 2 g that was devoid of fat and moisture. The samples were cooked in 1.25% H_2_SO_4_ for 30 min before being filtered and washed. These samples were then boiled in 1.25% NaOH for 30 min before filtering and washing. The residue was dried for two hours at 130°C before weighing. The dry residue was burned, cooled, and reweighed. The crude fiber was determined using the following formula given in Equation (6):

(6) $$\text{Crude Fiber}\%=\frac{\text{loss in weight}\;\mathrm{on}\;\text{ignition}-\text{Blank}\;}{\text{weight of Samples}}\times 100$$

#### 2.2.6 Nitrogen Free Extract (%)

It was estimated and obtained by employing the formula mentioned below in Equation (7):

(7) $$\mathrm{NFE}\;\left(\%\right)=100-\left(\%\text{Moisture}+\%\text{Crude protein}+\%\text{Crude}\;\mathrm{fat}+\%\text{Crude fiber}+\%\mathrm{Ash}\right)$$

### 2.3 Mineral Content

The AOAC (2005) method was used to determine the mineral contents of samples such as Na, K, Zn, Ca, Se, and Mg. At temperatures of up to 180°C–200°C, one gram of sample was digested with 10 mL of nitric acid: perchloric acid (7:3) combinations until translucent contents were produced. The mixture was diluted with double‐distilled water to a final amount of 100 mL. The concentration of mineral components was assessed by passing the diluted sample through an air‐acetylene flame‐powered Atomic Absorption Spectrophotometer (Model: Varian, AA‐240, VIC, Australia).

### 2.4 Extraction of Zn and Se

Ten grams of powdered mushroom samples or substrates were combined with 100 mL of 80% methanol. Samples were sonicated, shaken in a shaker (IKA‐Werke GmbH & Co. Kg, Staufen, Germany) for 8 h, centrifuged at 3000 rpm with a Universal 320 R centrifuge, and filtered through Whatman No. 4 paper. The extraction was performed twice, and the supernatants were combined and evaporated to dryness at 40°C using a Büchi Rotavapor R‐205 (Flawil, Switzerland). The leftovers were weighed and stored at −12°C until analysis. For further examination, the extract was redissolved in 1 mL of 80% methanol (Gąsecka et al. 2016).

### 2.5 Fortification of Zn and Se in the Rice Flour Cookies

Cookies were made with rice flour samples supplemented with Zn and Se extracted from shiitake mushroom at concentrations of 100, 150, and 200 mg per kg of total cookie dough according to the usual process outlined by (AACC 2000). Butter, sugar, salt, baking powder, egg, ghee, and water were used as common ingredients in all treatments, while Zn and Se extracted from shiitake powder were used as variable ingredients according to the treatments plan given in ST 1. All ingredients were weighed precisely and mixed homogeneously in a laboratory mixer bowl, Kenwood planetary mixer (Kenwood Ltd., Pakistan) for 10 min at 120 RPM speed for each treatment group (T_0_, T_1_, T_2_, and T_3_). After the homogenous mixing, a resting period of 10 to 15 min was applied. The dough was converted into a 5 mm thick sheet which was cut into cookies using suitable molds. Baking was carried out in a laboratory convection oven (Memmert, Pakistan) at 170°C for 12–15 min, followed by cooling at room temperature. After cooling, cookies were vacuum‐packed in propylene bags and stored at room temperature for 1–1.5 months for further analysis. However, each serving (one cookie) in groups T1, T2, and T3 provided an average of 10 mg:10 μg, 15 mg:15 μg, and 20 mg:20 μg of Zn and Se, respectively, as verified by formulation and batch testing. All batches were processed under standardized conditions to ensure ingredient homogeneity and batch‐to‐batch uniformity.

### 2.6 Efficacy Study

From Feb to July 2023, a single‐blind randomized control trial (in which participants were blinded) was conducted at an outpatient clinic for TB patients in a university hospital to investigate the effect of Zn and Se supplementation at various concentrations in cookies as an immune booster in Pulmonary Tuberculosis (Pul‐TB) patients. The Research Ethics Committee of The University of Lahore reviewed and approved this study on 10‐10‐2022 (REC‐UOL‐FAHS/906/2022), and all methods were performed in accordance with relevant institutional guidelines and regulations. Patients aged 30 to 55 with evident indications of Pul‐TB and verified positive acid‐fast bacilli in sputum samples were included in the study. Baseline characteristics of all participants, including age, sex, BMI, TB severity, nutritional status, and initial hematological parameters, were assessed and presented in ST 2. Patients who had other types of TB or had been on therapy for more than two months were excluded from the trial. All participants supplied informed written consent for study participation in accordance with the university's principles. Patients were excluded if they had renal dysfunction, malabsorption difficulties, or unstable weight in the three weeks or months before to the start of the trial. Pregnant patients were omitted, as were patients who refused to agree or did not attend the session. However, 120 TB infected patients were selected and randomly divided into four treatment groups (T0, T1, T2, T3). All three groups were given antituberculosis medications (a combination of ethambutol; 15 mg/kg of body weight (BW)), rifampicin (10 mg/kg of BW), isoniazid (5 mg/kg of BW), and pyrazinamide (25 mg/kg of BW) as recommended by WHO. The treatment technique used was direct observed treatment, short course (DOTS) in accordance with WHO standards. Moreover, to minimize food‐drug interactions, participants were given individualized dietary counseling and instructed to follow a standardized diet, avoiding zinc/selenium‐rich and high‐fat foods. Participants received personalized dietary counselling and were advised to adhere to a prescribed diet while avoiding foods that were especially high in zinc and selenium, as well as high‐fat meals in order to reduce potential food–drug interactions and dietary variability. Compliance was monitored using 24‐hour recalls and patient logbooks. Body mass index (BMI), microbiological, and hematological parameters were measured on a monthly basis, and biological parameters were measured for all patients at admission and at the end of the study. Chest radiography (CXR) was taken every fifteen days to assess the treatment's effectiveness on patients. Patients who had cavity lesions in their lungs had a decrease in the radius of the cavity. Microbiological investigations of sputum samples for the presence of MTB were performed every 15 days for a month of treatment and analyzed using ZN staining procedures. All the details are given in Table 1.

**TABLE 1:** Intervention and dosage details.

Groups		Patients	Treatment	Dosage	Follow‐up		
					1st day	15th day	30th day
Control group	(T_0_)	(n‐30 PTb)	Myrin P Fort	Rifampicin Isoniazid Pyrazinamide Ethambutol			
Placebo group	(T_1_)	(n‐30 PTb)	Zn + Se supplemented cookies	10 mg:10 μg			
	T2	(n‐30 PTb)	Zn + Se supplemented cookies	15 mg:15 μg			
Subject group	(T_3_)	(n‐30 PTb)	Zn + Se + Myrin P Fort	Rifampicin (300 mg) Isoniazid (300 mg) Pyrazinamide (500 mg) Ethambutol (100 mg) Cookies (20 mg:20 μg)			

*Note:* Control group: T0 = Myrin P Fort. Placebo group: T1 = Zn and Se enriched cookies (10:10); T2 = Zn and Se enriched cookies (15:15). Subject group: T3 = Zn and Se enriched cookies (20:20) + Myrin P Fort.

### 2.7 History of Patients

The initial screening was done in the labs (TB skin test & TB blood test, complete blood count with erythrocyte sedimentation rate, CBC/ESR, and CXR). Other symptoms of cough, chest pain, weight loss, dyspnoea, headache, fever, and night sweats were also monitored.

### 2.8 Anthropometric Measurements

Standardized procedures were used to measure BW and height by following the method (Nabi et al. 2024). The weighing and height scales were calibrated regularly, and all subjects were weighed while wearing minimal clothing. BMI was defined as weight in kilograms divided by the square of height in meters (kg/m^2^). The computed BMI of each patient was compared to the standard BMI reference table for Asians. The weight of the patients was determined by using a weighing balance. For height measurement, a scale was placed on a 6‐ft wall, and any additional inches were noted down. The mid‐arm circumference (MAC) and triceps skinfold muscle (TSF) were also assessed using the method described by Chen (2022).

### 2.9 Microbiological and Hematological Parameters

Blood samples from TB patients were collected via venipuncture, and various microbiological and hematological tests were performed, including ESR, total leucocytic count (TLC) (Patel et al. 2024).

### 2.10 Statistical Analysis

All resulting data were analyzed statistically to test the significance level (*p* ≤ 0.05) via the completely randomized design (CRD) through the analytical software Statistix (version 10.1), as described by Montgomery (2017). Moreover, Duncan's Multiple Range Test (DMRT) was used as a post hoc test to identify significant differences among treatments. Principal Component Analysis (PCA) and hierarchical clustering heatmaps were generated using R‐studio (Version 4.2.2) to examine relationships among attributes. PCA diminishes data dimensionality by generating independent linear combinations (principal components) that maximize variance, while heatmaps offer a color‐coded visualization of attribute correlations and clusters. These tools enhance the interpretation of multivariate data, making it easier to identify key trends and outliers, which supports more accurate decision‐making in food analysis.

## 3 Result and Discussion

### 3.1 Proximate Composition of Shiitake

Determining the nutritional composition or proximate composition of food is important for a variety of reasons, including analyzing the product's quality, safety, and nutritional worth. Physical and chemical changes in each constituent and ingredient result from processing operations and often lead to physical, chemical, sensory, and nutritional changes in the food (Galanakis 2021). Shiitake powder was subjected to proximate analysis, including moisture, ash, crude protein, and crude fiber before preparing the product. Average values of the moisture, fat, ash, crude protein, and crude fat obtained after the proximate analysis are shown in Table 2. The results of our current study were similar to the findings of Van Toan and Thu (2018), who determined the chemical composition of shiitake powder and utilized it in the preparation of cookies. Similarly, Li et al. (2021) conducted a comparative analysis of the nutritional value and chemical makeup of six cultivar strains of *L. edodes* that are well‐liked in China, and the results were in line with our current findings. The findings showed that all examined *L. edodes* had low levels of ash (5.24%–6.38%) and low levels of fat (0.80%–1.70%). They were also good sources of protein (14.87%–27.13%), carbs (62.03%–75.56%), and dietary fiber (35.88%–42.49%). However, significant variations existed among shiitake mushroom (*L. edodes*) cultivars primarily due to genetic diversity (both in cultivated and wild strains), breeding efforts to create specific traits, and diverse environmental/cultivation factors.

**TABLE 2:** Chemical composition of Shiitake.

Constituents	Chemical composition
Moisture %	83.9 ± 0.7
Ash %	19.6 ± 0.9
Protein %	27.4 ± 0.7
Fat %	3.7 ± 0.4
Fiber %	14.7 ± 0.8
NFE %	34.5 ± 0.9
Energy (kcal)	252.6 ± 0.5
Calcium (mg/kg)	232.8 ± 1.2
Sodium (mg/kg)	700.7 ± 1.9
Iron (mg/kg)	17.6 ± 0.6
Zinc (mg/kg)	15.8 ± 0.4
Selenium (mg/kg)	7.4 ± 0.01

*Note:* Values are expressed as mean ± standard deviation (*n* = 3).

### 3.2 Mineral Content

Minerals are a necessary component of life and can be obtained from food sources. Essential minerals are inorganic nutrients that are required by the human body for a variety of physiological activities (Malik et al. 2023). Minerals are essential for establishing and preserving excellent health. A highly significant (*p* ≤ 0.01) result for mineral analysis of edible mushrooms (species shiitake) was observed in our current study. However, mean values for minerals calcium (Ca), sodium (Na), iron (Fe), Zn, and Se in shiitake are shown in Table 2. Na is an essential mineral for both cellular and electrical functions in the body and is involved in maintaining the ionic balance of the human body. By reducing the effects of Na and encouraging healthy blood vessel function, potassium (K) helps to control blood pressure. However, mushrooms are also a significant source of Ca that is necessary to develop and maintain healthy bones and teeth and are needed for the regulation of muscle contraction, glandular secretion, and for mediating vascular contraction.

Additionally, it affects blood coagulation and muscle function (Tang et al. 2023). Zn and Se are essential minerals that play important roles in the body's immune system and overall health. Zn is crucial for a well‐functioning immune system. It is involved in immune cell development and function. Se acts as an antioxidant, helping to protect cells from oxidative damage. Through the overproduction of reactive oxygen species (ROS), tuberculosis causes oxidative stress, which raises lipid peroxidation and lowers antioxidant defenses like glutathione, superoxide dismutase, and catalase (Shastri et al. 2018). By serving as cofactors for antioxidant enzymes and boosting glutathione‐dependent defense mechanisms, zinc and selenium help restore redox balance and lessen oxidative damage in TB patients. A diet rich in selenium‐containing foods or selenium supplementation may be beneficial for patients with tuberculosis because selenium plays an important role in antioxidant defense. Previously, Li et al. (2021) investigated the mineral content in shiitake and the results were in line with our current findings. In another research study conducted by Ho et al. (2020) in which shiitake was utilized for the development of muffins and characterized for the determination of biochemical and mineral profile and results indicated the presence of magnesium (Mg), K, phosphorus (P), Na, and Zn. Thus, the results obtained were similar to our recent study. However, George et al. (2014) determined the major and minor mineral elements level through a comparative analysis from the mycelia and the fruiting bodies of shiitake and results showed that in addition to substantial concentrations of Zn, Fe, Cu, manganese (Mn), lead (Pb), and nickel (Ni), the roots of fungi included high levels of Na, K, and P. The principal elements found in the fungi's visible portion, the fruiting bodies, were K, P, and Mg. There were also significant amounts of Zn, Fe, Mn, and Cu. These results are in line with our findings, which show that shiitake mushrooms have significant quantities of important minerals, supporting their potential as a nutritious component of food items.

### 3.3 Product Development

As previously discussed, shiitake contains 15.8 mg of Zn and 7.4 mg of Se; thus, when compared to the control cookies, the Se and Zn content of the tested cookie samples increased, whereas other contents did not differ in all cookie samples, and the results were non‐significant. The fortified cookies contained standardized Zn and Se levels ranging from 10 ± 0.3 to 20 ± 0.4 mg and 10 ± 0.05 to 20 ± 0.07 μg per serving, confirming exact dosages and batch uniformity. Fortification is the deliberate increase of one or more micronutrients (such as vitamins and minerals). When compared to earlier investigations, cookies baked with rice flour alone had better organoleptic properties, with no significant difference between the control sample and the fortified specimens containing 10, 15, 20, and 25 mg of Zn and Se. For cookies containing 25 mg of Zn and Se, the organoleptic qualities indicated substantial changes in scores of all sensory properties except taste when compared to the control sample and other fortified samples. Furthermore, cookies were given to TB patients to test the bioactivity of Zn and Se on TB immune modulatory characteristics.

### 3.4 History and Clinical Evaluation of Patients

The results of the history and clinical evaluation of the patients revealed that the majority of TB patients (97%) had a cough for the last three weeks, approximately 96% of the subjects had a high fever at night. Other symptoms observed were hemoptysis, malaise, tiredness, anorexia, chest pain, and night sweating with relative frequencies of 9%, 48%, 45%, 65%, 59%, and 48%, respectively as shown in ST 3.

### 3.5 Anthropometric Measurements

Anthropometric measurements, which are physical traits and measurements of the human body, can be significantly influenced by nutritional supplements. These metrics are frequently used to examine body composition and overall health changes. The effect of nutritional supplementation on anthropometric measurements varies depending on the type of supplementation, the individual's baseline health and nutritional state, and their general diet and lifestyle (Madden and Smith 2016). Nutritional supplements, however, shouldn't be used as the main tuberculosis treatment. Alternatively, they could be used in conjunction with standard anti‐tuberculosis drugs as supportive therapy to enhance recovery and nutritional status.

#### 3.5.1 Weight

**FIGURE 1:**
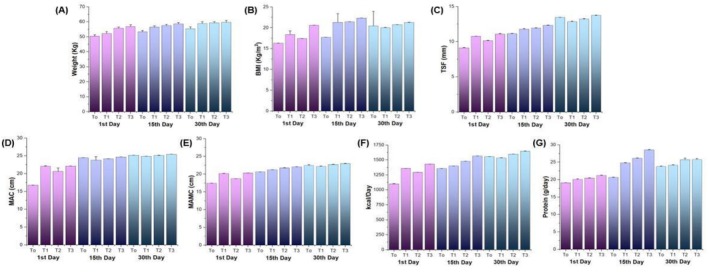
Graphical representation of mean ± standard deviation (SD) illustrating the effect of Zn and Se supplementation on anthropometric and dietary parameters across treatment groups: T0 (Control), T1 (10:10), T2 (15:15), and T3 (20:20), assessed at 1st, 15th, and 30th day follow‐ups. Panels represent: (A) Weight (kg), (B) Body Mass Index (BMI, kg/m²), (C) Triceps Skinfold Thickness (TSF, mm), (D) Mid‐Upper Arm Circumference (MAC, cm), (E) Mid‐Arm Muscle Circumference (MAMC, cm), (F) Energy intake (kcal/day), and (G) Protein intake (g/day).

Wasting is a systemic clinical symptom of TB that can impact the progression of illness and severity. Terms such as “phthisis” and “consumption” indicate the historical association between TB and body wasting. With variable reasons for wasting, including changes in appetite and metabolism, the link between infection and nutrition is complex. Although weight gain is frequently utilized to evaluate the effectiveness of treatment, its relevance and efficacy as a forecaster of clinical results in TB patients are still up for debate. The descriptive data of the study's subjects (*n* = 120), and the mean results showed that there were significant differences (*p* ≤ 0.05) between the weight of patients among all treatments T_0_, T_1_, T_2_, and T_3_ on the 1st day, 15th day and 30th day as shown in Figure 1. The inflammatory process can be closely linked to weight loss; thus, the results showed that on the first day, the weight of the patients decreased and gradually improved with proper administration of Zn and Se‐based diets (fortified cookies) as shown in follow‐ups at the 15th and 30th day. Therefore, dietary supplementation with Zn and Se increases the total intake of energy and causes weight gain with only slight suppression of voluntary intake of ordinary food, which also helps to fight and support the immune system as well as allow more functional and quality of life. In a previous study, Abba et al. (2008) and Wagnew et al. (2022) examined the impact of zinc supplementation on the results of TB treatment. Results showed that after six weeks, underweight TB patients who were given high‐energy nutritional supplements gained noticeably more weight than the control group. Therefore, individuals with TB who consume cookies enriched with Zn and Se may benefit from improved anthropometric measurements and overall health. Malnutrition, as shown by decreased BW and body fat percentage (BFP), is a common occurrence in children with TB. In another study, Mexitalia et al. (2017) observed increased BW, BFP, and leptin levels in a study on TB‐affected children receiving anti‐TB treatment; these findings suggested that the children had responded favorably to both the intensive and continuation phases of treatment. To compare these results with data from healthy youngsters, more research is required.

#### 3.5.2 BMI Calculation

BMI is frequently used to evaluate the nutritional condition of TB patients; however, studies typically classify it either continuously or using a single cut‐off point, which is typically 18.5 kg/m^2^. Among TB patients, underweight frequency is three times higher, and there is a correlation between poor nutritional status and higher TB severity and death rates. According to an Indian study, there is a 22% decrease in mortality for every unit increase in baseline BMI (Singh et al. 2022). Few studies provide insight at the relation between overweight/obesity and TB risk or mortality in developing countries, despite the increasing amount of research on the association between nutrition and tuberculosis. To better understand how excess body weight affects TB outcomes in these populations, more research is therefore required. Research conducted in a variety of groups demonstrates that the incidence of TB and BMI are inversely correlated, but nothing is known about the relationship between treatment outcomes and overweight. With consideration for gender differences in clinical presentation and results, our study attempts to evaluate the effects of underweight and overweight on anti‐TB treatment outcomes. There are notable differences in the geographic distribution and severity of TB between the sexes. There is still insufficient research on the effect of nutrition on sex‐based TB outcomes. We predict that on the 1st, 15th, and 30th days of follow‐up, men and women experience different effects of nutritional status, specifically Zn and Se from shiitake at baseline, on anti‐TB treatment outcomes. The findings were shown in Figure 1, which demonstrates that highly significant differences (*p* ≤ 0.01) were found in all treatments. While the effect of experimental cookies prepared by different concentrations of Zn and Se on the BMI of TB patients was shown to give a significant effect at 30th day follow‐up. Previously, Ren et al. (2019) examined dietary intakes and found risk variables for 300 adult TB patients living in underdeveloped areas of China, and the results of this study were in line with our current findings. Males (1655.0 kcal) and females (1360.3 kcal) did not meet the necessary daily energy intake, with 87.4% of males and 59.9% of females not achieving the needed daily energy intake. Additionally, protein intake was deficient, as 90.8% of men and 58.4% of women reported low daily protein intakes. In order to improve the dietary adequacy and health outcomes of TB patients in underdeveloped regions, targeted nutritional interventions and support are crucial. This is demonstrated by the correlation between low energy intake and unemployment, as well as the connection between higher protein consumption and eating out.

#### 3.5.3 Determination of MAC, MAMC, and TSF

A portable and easy‐to‐use way to measure muscle mass is mid‐arm muscle circumference (MAMC), which is computed as MAC minus 0.3142 times TS thickness. It is essential to comprehend commonly used anthropometric indices, such as MAMC, in order to create targeted preventative and treatment plans for a range of diseases. While TSF evaluates BFP in conjunction with other anthropometric data, MAMC shows lean body mass and nutritional health (Mohmmad et al. 2024). Assessing total arm circumference is useful in determining changes in body composition and nutritional status. Cookies prepared with rice flour enriched with Zn and Se contents extracted from shiitake exhibit favorable responses on mineral management in the body, particularly during TB. However, the statistical results showed that there were significant differences (p ≤ 0.05) between the MAMC, MAC, and TSF of patients among all treatments T_0_, T_1_, T_2_, and T_3_ and shown in Figure 1. A cross‐sectional study was carried out by Sharma et al. (2021) on 251 patients with cirrhosis to determine the prevalence of malnutrition and the factors influencing nutritional consumption and the results were similar to our current investigations. A total of 65% of patients had malnutrition, with 35% classified as well‐nourished, 42% as moderately nourished, and 23% classified as severely malnourished. Those who were well‐nourished consumed much more calories and protein and measured their midarms higher than those who were moderately or severely underweight. Poor appetite, early satiety, abdominal fullness, low‐salt diets, and societal diet beliefs were among the variables affecting food consumption. In another research, Sharma et al. (2021) compared nutritional assessments in patients with multidrug‐resistant TB (MDR‐TB), human immunodeficiency virus TB (HIV‐TB), and Pul‐TB. CXR showed minimum disease in most HIV‐TB patients, moderately advanced disease in most MDR‐TB patients, and minimal disease in Pul‐TB patients. TSF revealed undernutrition in all patients, with notable variations between the HIV‐TB and MDR‐TB groups. The multifactorial impact of nutritional deficiencies on immune function, treatment response, and disease progression in TB patients was emphasized even more by the study's findings that lifestyle factors like smoking and longer disease duration were significantly correlated with changes in biochemical parameters (such as serum protein, hemoglobin) and anthropometric measures.

#### 3.5.4 Calorie Count

MTB infection increases the risk of malnutrition, and malnutrition itself is a result of TB infection. An elevated basal metabolism brought on by a TB infection causes the body to require 10%–30% more energy, which might cause an imbalance in the production and breakdown of proteins. Free fat mass is lost as a result of this imbalance, which causes protein stores to be depleted. For TB patients, the recommended daily protein intake is 1.2–1.5 g/kg BW, and the recommended energy requirement is 35–40 kcal/kg BW (Yunda et al. 2020). According to our current research, the nutritional status of all patients (*n* = 120) has varying effects on anti‐TB therapy results on the first, fifteenth, and thirtieth days of follow‐up. The results are displayed below in Figure 1, which shows that there were significant differences (*p* ≤ 0.05) in every treatment. However, the results were in accordance with the previous findings of Andrade and Garcia‐Perdomo (2020), who conducted a systematic evaluation of 16 randomized controlled trials with 4398 participants to examine the effect of micronutrients on the prevention and treatment of Pul‐TB. When given, Zn boosted weight gain (Mean difference (MD), 3.10) and muscle mass index (MD, 1.20), while decreasing mortality (Risk difference (RD), 0.04). Weight and the Karnofsky scale significantly improved when Zn and vitamin A were combined. Sputum conversion time was speeded up by vitamin D, and significant alterations were observed when combined with hemoglobin, vitamin A, and Zn. Micronutrient supplementation showed improvements in sputum conversion, hemoglobin, weight gain, and quality of life; nevertheless, bigger studies with optimal doses are recommended for assessing mortality. In a prospective cohort study, Feleke et al. (2019) evaluated micronutrient deficits in patients with TB at the beginning and conclusion of the intense phase. Thus, initial observations indicated substantial nutritional deficiencies; however, the integration of antituberculosis therapy with nutrient‐enriched diets significantly enhanced patient recovery and treatment outcomes.

#### 3.5.5 PCA of Anthropometric Measurements

**FIGURE 2:**
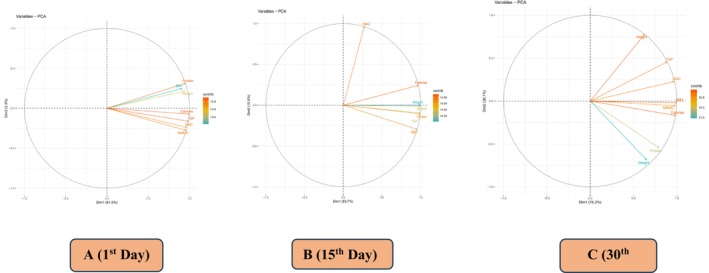
Principal component analysis (PCA) plot illustrating the effect of Zn and Se supplementation on anthropometric measurements and the association among all treatment groups T_0_ = Control; T_1_ = (10:10); T_2_ = (15:15); T_3_ = (20:20) at 1st, 15th, and 30th day follow up among groups (T_0_, T_1_, T_2_, T_3_). A (1st day follow up); B (15th day follow up); C (30th day follow up).

The loading plots given in Figure 2 of PCA showed how nutrients such as Zn and Se affect weight, BMI, TSF, MAC, MAMC, protein, and energy, as well as how they contribute to the two main dimensions (Dim1 and Dim2) of variance. While in Figure 2A, Dim2 accounts for 5.3% of the variation, Dim1 accounts for 91.5%, indicating its significance in the analysis during 1st day follow‐up of the study. Nevertheless, Dim1 accounts for 53.7% of the variance in Figure 2B, emphasizing its significance in the analysis, while Dim2 accounts for 15.61% during the follow‐up period of 15 days. While on the 30th day, follow‐up variables like height, TSF, MAC, BMI, MAMC, calories, protein, and weight are plotted against two principal dimensions (Dim1 and Dim2) in Figure 2C. Dim1 accounts for 76.2% of the variance, emphasizing the major trends in the dataset, while Dim2 explains 20.7% of the variance, highlighting additional, less dominant patterns. It is possibly the goal of this investigation to determine correlations over time between body composition, physical measurements, and dietary consumption. Understanding the relative significance or influence of different nutrients in the dataset under analysis is made easier by this visualization. The fact that all investigated parameters were successfully distributed throughout the database indicates that Se and Zn had a significant impact on the anthropometric measures and energy level of TB patients.

#### 3.5.6 Heat Map

**FIGURE 3:**
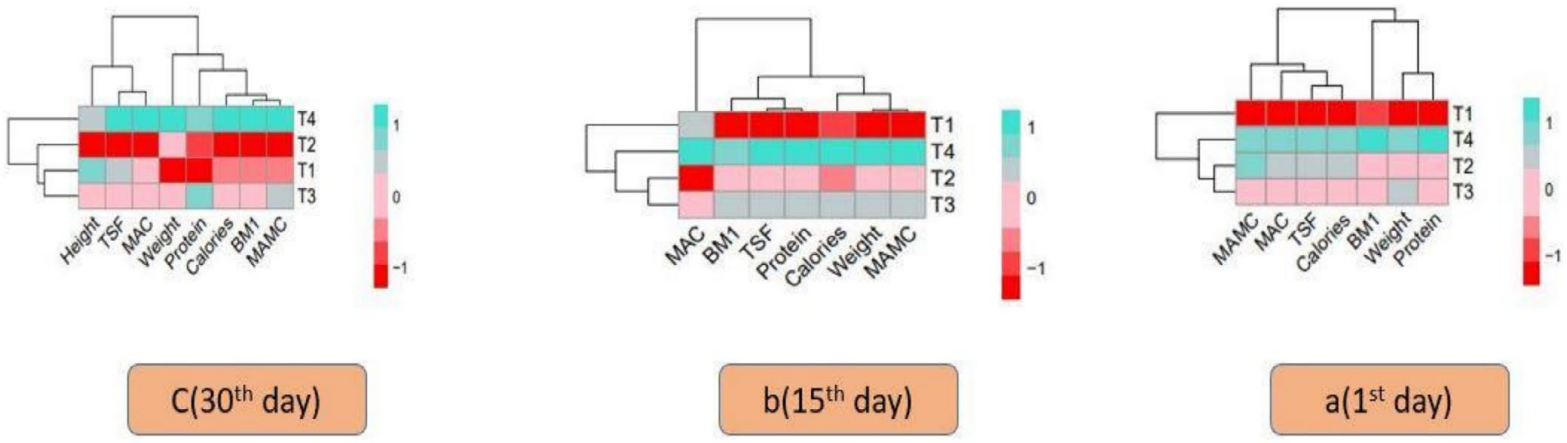
Heat map visualization of correlation between anthropometric variables and among all treatment groups T_0_ = Control; T_1_ = (10:10); T_2_ = (15:15); T_3_ = (20:20) at 1st, 15th, and 30th day follow up. A (1st day follow up); B (15th day follow up); C (30th day follow up).

Histogram correlation analysis was used to show how different treatments related to TSF, MAC, BMI, (MAMC), calories, protein, and weight. The results are shown in Figure 3. In the histogram study, purple, green, and red colors indicate a significant difference, while blue color indicates non‐significant changes within the treatments. The histogram clearly illustrates the differences between therapies T_0_, T_1_, T_2_, and T_3_ for TB patients with varying Zn and Se concentrations.

### 3.6 Microbiological Parameters

**FIGURE 4:**
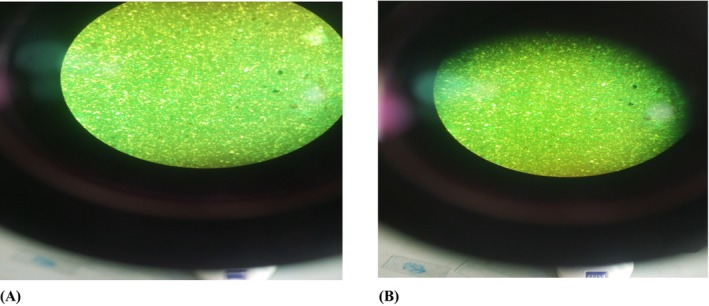
(A) Acid Fast Bacilli load (AFB) at 15th day. (B) Acid Fast Bacilli load (AFB) at 30th day.

Ultimately, the microbiological evaluation of TB patients using Myrin Fort with Zn and Se is useful in optimizing treatment regimens, maintaining patient well‐being, and furthering our understanding of TB therapy on both an individual and public health level. For this purpose, Sputum smear microscopy (SSM) was used and mainly performed in peripheral microscopy centers through the Hot Ziehl‐Neelsen technique and two consecutive early morning sputum samples (5–10 mL) per participant, obtained by spontaneous coughing effort and 240 sputa from 120 participants. The microbiological evaluation of patients with HIV receiving Myrin Fort, which is fortified with Zn and Se, was determined and the results of each follow‐up are shown in Table 3 below. Mean results for T_0_, T_1_, T_2_, and T_3_ at the 1st day and 15th follow‐up showed positive results and MTB as well as Rifampicin resistance were detected during the XPERT MTB/RIF assay. However, during follow‐up at the 30th day mean results for T_0_, T_1_, T_2_, and T_3_ showed negative results and lower MTB with no resistance to Rifampicin were determined during the XPERT MTB/RIF assay as represented in Figure 4A,B below.

**TABLE 3:** Sputum samples for AFB load.

Follow‐up	Groups	Specimen for AFB stain		Positive	Negative
		Septum	Non septum		
1st day	T0 (n‐30)	(7 mL)	—	3+	—
	T1 (n‐30)	(7 mL)	—	3+	—
	T2 (n‐30)	(7 mL)	—	3+	—
	T3 (n‐30)	(7 mL)	—	3+	—
15th day	T0	(7 mL)	—	2+	—
	T1	(7 mL)	—	2+	—
	T2	(7 mL)	—	2+	—
	T3		(2 mL)	2+	—
30th day	T0	(5 mL)	—	1+	
	T1	—	(5 mL)		Neg
	T2	—	(5 mL)		Neg
	T3	—	(5 mL)		Neg

*Note:* Values are expressed as Mean ± Standard Deviation (*n* = 3). Control group: T0 = Myrin P Fort. Placebo group: T1 = Zn and Se enriched cookies (10:10); T2 = Zn and Se enriched cookies (15:15). Subject group: T3 = Zn and Se enriched cookies (20:20) + Myrin P Fort.

Nevertheless, Zn protects cells from the damaging effects of free radicals and in the contribution of macrophages' host defense, limiting membrane damage during inflammation. In a previously published meta‐analysis of randomized controlled trials, Hussain et al. (2019) described the immune modulatory and anti‐oxidative effect of Se for TB and the results were in accordance with our current findings. Thus, reduced inflammation, facilitated by micro‐nutrient support, especially Zn and Se, may enhance the effectiveness of TB treatment, as evidenced by improved sputum smears and culture conversions, suggesting better disease control and treatment success.

### 3.7 Hematological Analysis

**FIGURE 5:**
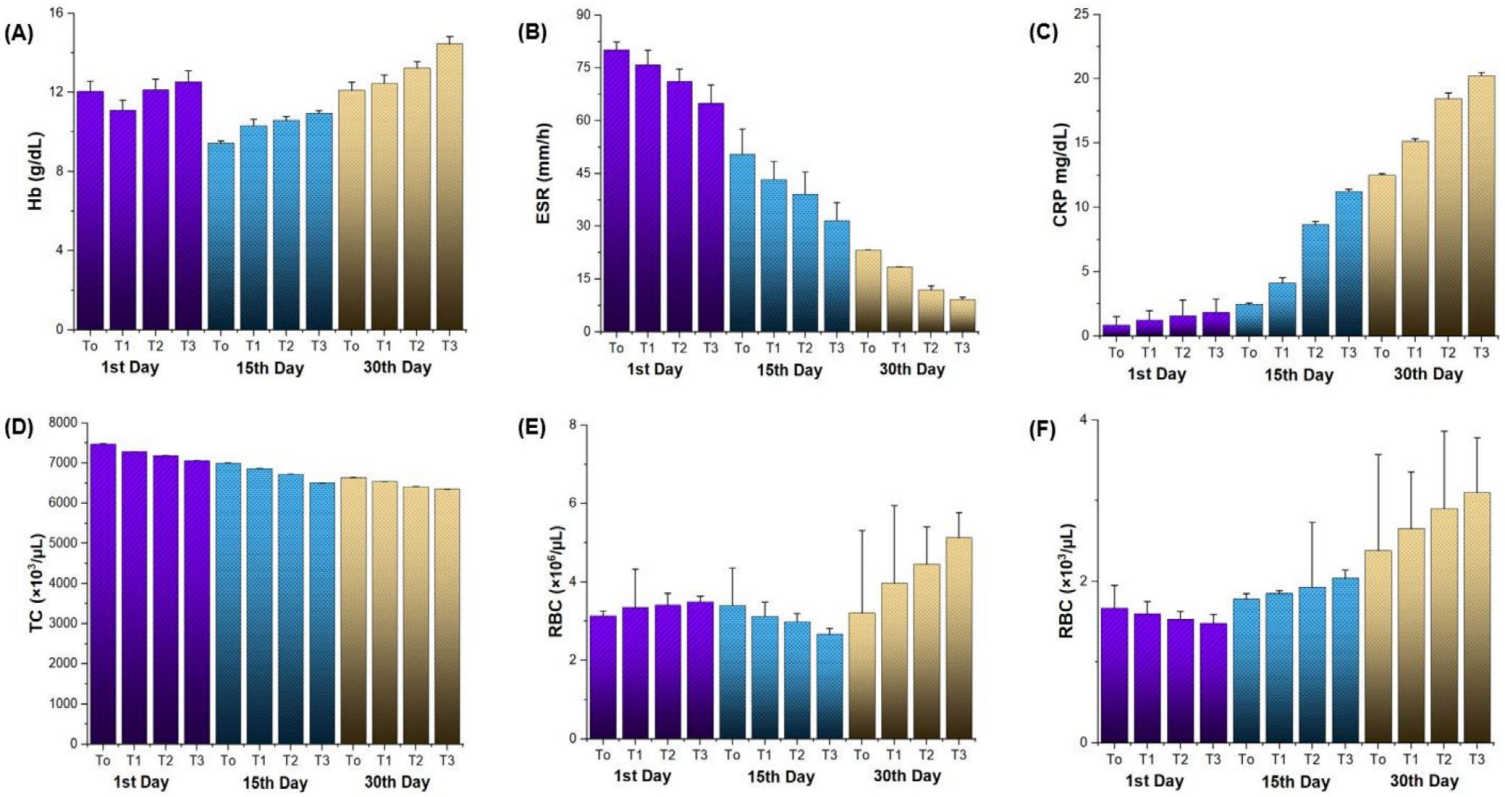
Graphical representation of mean ± standard deviation (SD) illustrating the effect of Zn and Se supplementation on hematological parameters across treatment groups: T0 (Control), T1 (10:10), T2 (15:15), and T3 (20:20), assessed at 1st, 15th, and 30th day follow‐ups. Panels represent: (A) Hemoglobin (Hb, g/dL), (B) Erythrocyte Sedimentation Rate (ESR, mm/h), (C) C‐Reactive Protein (CRP, mg/dL), (D) Total Cholesterol (TC, ×10³/µL), (E) Red Blood Cells (RBC, ×10^6^/µL), and (F) White Blood Cells (WBC, ×10³/µL).

Hematological analysis is the process of analyzing blood components to evaluate different aspects of health. It covers the investigation of plasma components and blood cells. The study of blood cellular components, including red blood cells (erythrocytes), white blood cells (leucocytes), and platelets (thrombocytes), as well as how these data are used to diagnose and track disease, is known as hematology. Hematological analysis is critical for tracking the progression of TB. Changes in white blood cell counts, particularly lymphocytes and neutrophils, can reveal information about the body's reaction to infection (Iqbal et al. 2015). The descriptive data of the study's subjects (*n* = 120) are shown in Figure 5. The statistical findings demonstrated that there were significant variations (*p* ≤ 0.05) in the patient's Hemoglobin test (Hb), ESR, C‐reactive protein (CRP), Total count (TC), Red blood cells (RBC), and platelets throughout all treatments (T_0_, T_1_, T_2_, and T_3_) as shown in Figure 5. Anemia is a common hematologic complication among TB patients and a significant risk factor for mortality. However, in our current investigation, mean results showed that the Hb level at the onset of TB decreased considerably, leading to chronic inflammation and improved from 10 to 14 g/dL after consuming Zn and Se‐supplemented cookies for 30 days in a randomized control trial. ESR is part of a well‐established routine investigation for TB, which showed the highest mean values (80.2 ± 2.1) at the 1st day of follow‐up while on regular consumption of Zn and Se fortified cookies; inflammation in the body reduced and showed (9.1 ± 0.7) mean values at the 30th day of follow up. In our study, both the micro‐nutrient support group and the control group experienced reductions in leukocyte, neutrophil, CRP, and ESR counts, along with increased lymphocyte counts in response to MTB. Notably, the micro‐nutrient group exhibited a more pronounced effect.

Meanwhile, WBCs rise during infection as a result of increased polymorphonuclear leukocytes and macrophages, which are part of the body's immune defense strategy to resist the invading germs. During anti‐TB treatment, lymphocyte counts initially decreased and then increased, while leukocyte and neutrophil counts showed opposite trends. In another study, Singh et al. (2013) reported the effect of vitamins and minerals on TB patients and the results were in accordance with our current outcomes.

#### 3.7.1 PCA of Hematological Analysis

**FIGURE 6:**
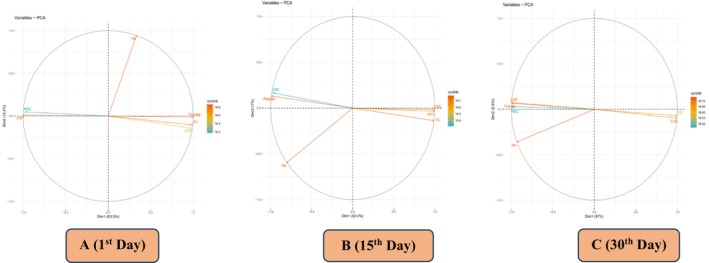
Principal component analysis (PCA) plot illustrating the effect of Zn and Se supplementation on hematological measurements and the association among all treatment groups T_0_ = Control; T_1_ = (10:10); T_2_ = (15:15); T_3_ = (20:20) at 1st, 15th, and 30th day follow up among groups (T_0_, T_1_, T_2_, T_3_). A (1st day follow up); B (15th day follow up); C (30th day follow up).

PCA loading plots provided in Figure 6 illustrate how nutrients like Zn and Se impact HB, ESR, CRP, TC, platelets, and RBC in addition to their contributions to the two primary dimensions (Dim1 and Dim2) of variance. While Dim2 accounts for 15.4% of the variation in Figure 6A, Dim1 accounts for 83.5%, suggesting that Dim1 is significant in the analysis conducted during the study's first day of follow‐up. However, over the 15‐day follow‐up period, Dim2 accounts for 7% of the variance, while Dim1 accounts for 92.4% of the variance in Figure 6B, highlighting its significance in the analysis. On the other hand, Figure 6C showed the follow‐up variables at day 30 plotted against the two main dimensions (Dim1 and Dim2). While Dim2 explains 2.6% of the variation and highlights supplementary, less prominent patterns, Dim1 accounts for 97% of the variance and highlights the primary trends in the dataset. Determining relationships between clinical data and hematological markers throughout time may be the aim of this study. This visualization facilitates the interpretation of the relative importance or influence of various nutrients in the dataset under examination. The successful distribution of all examined parameters across the database suggests that Zn and Se significantly influenced the hematological measurements of TB patients.

#### 3.7.2 Heat Map

**FIGURE 7:**
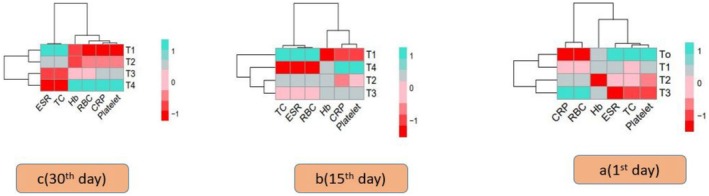
Heat map visualization of correlation between hematological variables and among all treatment groups T_0_ = Control; T_1_ = (10:10); T_2_ = (15:15); T_3_ = (20:20) at 1st, 15th, and 30th day follow up. A (1st day follow up); B (15th day follow up); C (30th day follow up).

Hematological data, including Hb, ESR, CRP, TC, platelets, and RBC, were correlated with various therapies using histogram correlation analysis and findings were displayed in Figure 7. Purple, green, and red in the histogram research denote a significant difference, whilst blue shows non‐significant variations within the treatments. The disparities between therapies T_0_, T_1_, T_2_, and T_3_ for TB patients with different Zn and Se contents are clearly shown in the histogram.

## 4 Conclusion

This study showed the potential of Zn and Se supplementation extracted from shiitake mushroom to enhance the immunomodulatory response in TB patients, with results indicating significant improvements in anthropometric, hematological and microbiological parameters over 30 days period. The fortified cookies with Zn and Se, when combined with antituberculosis therapy, significantly modulated the immunological markers by reducing inflammatory indicators such as CRP and ESR while improving hematological profiles, including increased hemoglobin and RBC counts, indicating enhanced immune response during TB treatment. Furthermore, the antioxidant and anti‐inflammatory properties of Zn and Se showed an ameliorative effect on immune modulation, improving TB treatment outcomes without any reported adverse effects, including gastrointestinal disturbances (nausea, vomiting, abdominal pain), supporting their safety and effectiveness. These findings suggest that integrating Zn and Se supplementation into TB treatment protocols could enhance immune function, aid in recovery, and potentially reduce antibiotic resistance. Further research is recommended to explore the long‐term effects of this approach on diverse TB patient populations.

## Author Contributions

**Hanif Mughal:** conceptualization, methodology, data interpretation, and writing original draft. **Tabussam Tufail:** writing review and editing and supervision. **Huma Bader ul Ain:** methodology. **Amara Rasheed:** software. **Esther Ugo Alum:** supervision and resources.

## Funding

The authors have nothing to report.

## Disclosure

*Declaration of AI Use*: No generative artificial intelligence (AI) tools were used in the writing, data analysis, or figure preparation of this manuscript. All content was developed and verified entirely by the authors.

## Ethics Statement

A single‐blind randomized control trial was carried out to determine the effect of zinc and selenium on the immunomodulatory parameters of tuberculosis approved by the Ethics Committee of The University of Lahore on 10‐10‐2022 (REC‐UOL‐FAHS/906/2022).

## Conflicts of Interest

The authors declare no conflicts of interest.

## Data Availability

The data that support the findings of this study are available on request from the corresponding author. The data are not publicly available due to privacy or ethical restrictions.
